# A new paramagnetically shifted imaging probe for MRI

**DOI:** 10.1002/mrm.26185

**Published:** 2016-02-28

**Authors:** P. Kanthi Senanayake, Nicola J. Rogers, Katie‐Louise N.A. Finney, Peter Harvey, Alexander M. Funk, J. Ian Wilson, Dara O'Hogain, Ross Maxwell, David Parker, Andrew M. Blamire

**Affiliations:** ^1^ Dept. of ChemistryDurham UniversitySouth RoadDurhamUnited Kingdom; ^2^ Northern Institute for Cancer ResearchNewcastle UniversityUnited Kingdom; ^3^ Institute of Cellular Medicine & Newcastle MR CentreNewcastle UniversityUnited Kingdom

**Keywords:** contrast agent, molecular imaging, paramagnetic shift, temperature mapping

## Abstract

**Purpose:**

To develop and characterize a new paramagnetic contrast agent for molecular imaging by MRI.

**Methods:**

A contrast agent was developed for direct MRI detection through the paramagnetically shifted proton magnetic resonances of two chemically equivalent *tert*‐butyl reporter groups within a dysprosium(III) complex. The complex was characterized in phantoms and imaged in physiologically intact mice at 7 Tesla (T) using three‐dimensional (3D) gradient echo and spectroscopic imaging (MRSI) sequences to measure spatial distribution and signal frequency.

**Results:**

The reporter protons reside ∼6.5 Å from the paramagnetic center, resulting in fast *T*_1_ relaxation (*T*_1_ = 8 ms) and a large paramagnetic frequency shift exceeding 60 ppm. Fast relaxation allowed short scan repetition times with high excitation flip angle, resulting in high sensitivity. The large dipolar shift allowed direct frequency selective excitation and acquisition of the dysprosium(III) complex, independent of the tissue water signal. The biokinetics of the complex were followed in vivo with a temporal resolution of 62 s following a single, low‐dose intravenous injection. The lower concentration limit for detection was ∼23 μM. Through MRSI, the temperature dependence of the paramagnetic shift (0.28 ppm.K^−1^) was exploited to examine tissue temperature variation.

**Conclusions:**

These data demonstrate a new MRI agent with the potential for physiological monitoring by MRI. Magn Reson Med 77:1307–1317, 2017. © 2016 The Authors Magnetic Resonance in Medicine published by Wiley Periodicals, Inc. on behalf of International Society for Magnetic Resonance in Medicine. This is an open access article under the terms of the Creative Commons Attribution License, which permits use, distribution and reproduction in any medium, provided the original work is properly cited.

## INTRODUCTION

Contrast agents are routinely used to improve the diagnostic specificity of MRI. The most common agents use chelated gadolinium in which the local dipolar field of the Gd ion causes an increase in the longitudinal relaxation rate (*R*_1_) of water molecules within the local vicinity (and can increase tissue $R_{2}^{*}$ through local susceptibility effects). The presence of the contrast agent is inferred from the resulting intensity change on *T*_1_ or $T_{2}^{*}$ weighted MRI, respectively. These contrast systems are entirely passive, accumulating and being removed from tissues by diffusive processes only. Regionally differing contrast enhancement arises from local rates of delivery, accumulation, and clearance 1. A new frontier for MRI contrast agent design is to create functionalized probes that target specific cellular or physiological properties of the disease under investigation. Agents that bind to a range of targets, such as collagen in fibrotic scar tissue 2, 3, 4 or the endothelial wall 5, 6 have been developed. All of these agents also rely on indirect detection via changes in water relaxation rates (*R*_1_, *R*_2_, or $R_{2}^{*}$) through conventional gadolinium chelates 1 or iron oxide systems 7, rather than detecting the contrast molecule directly.

The presence of the lanthanide metal within a contrast agent acts on nuclei in the local structure of the chelating molecule and can induce large paramagnetic shifts, yielding distinct resonances that can be detected, offering the possibility of directly detecting the agents themselves. Several studies have examined this effect as a mechanism for molecular imaging, typically using thulium complexes (TmDOTA^–^ or TmDOTMA^–^). In these cases, proton groups within the DOTA or DOTMA structures are paramagnetically shifted by tens to hundreds of parts per million from the water signal, and therefore lie far beyond the biological proton resonant frequency range. Frequency selective acquisition can then be used to detect these signals independent of the main water peak 8, 9, 10, 11. The magnitude of the paramagnetic shift is sensitive to physiological conditions including temperature 10, 11, 12 and pH 12, 13, conveying functionality to these molecules. Previous studies have demonstrated the feasibility of in vitro 10, 11 and in vivo 8, 9, 10, 14, 15, 16, 17, 18, 19 molecular imaging of these agents, and highlighted the challenge in obtaining sufficient sensitivity, with most studies using high‐dose intravenous injection (5–10 times the clinical contrast dose) and renal ligation to prevent clearance of contrast from blood and sustain an artificially enhanced tissue and vascular concentration.

Certain studies have explored the use of different lanthanide metals 9, 11, but have not sought to optimize the chelator to both maximize reporter group structure and optimize relaxation properties, which are essential factors in defining overall measurement sensitivity. We have previously developed a small molecular weight ^19^F‐labelled lanthanide metal chelate, in which the structure was manipulated such that the dipolar field of the metal enhanced the longitudinal relaxation rate of the ^19^F nuclei. Increasing *R*_1_ allowed rapid pulsing in the MR experiment, lowering the detection threshold to approximately 20 μM of complex 20, 21. Here, we apply the same principles of lanthanide enhanced relaxation and design a new chelate with two chemically equivalent proton reporter groups to provide favorable operating conditions for high‐sensitivity molecular MRI, while retaining the extremely large paramagnetic shift of the proton reporter group. The detected MR signal from the reporter groups is outside the biological proton resonant frequency range, allowing three‐dimensional (3D) imaging of the molecular probe against zero background. As an exemplar of using this type of agent as a physiological probe, regional tissue temperature variation was determined in vivo using MR spectroscopic imaging. To distinguish this direct detection approach from other indirect MR detection methods of similar lanthanide probes (eg, PARACEST) 22, we previously suggested the term PARASHIFT agents when describing such lanthanide MR contrast agents 23.

## METHODS

This study was conducted in two parts. First, the PARASHIFT agents were synthesized and NMR relaxation properties measured to determine the structural characteristics of the complex. Second, the relaxation data were used to define the optimal scanning conditions, and in vivo imaging, biodistribution analysis, and tissue temperature mapping studies were undertaken in mice.

### Synthesis and Characterization of PARASHIFT Agents

**Figure 1:**
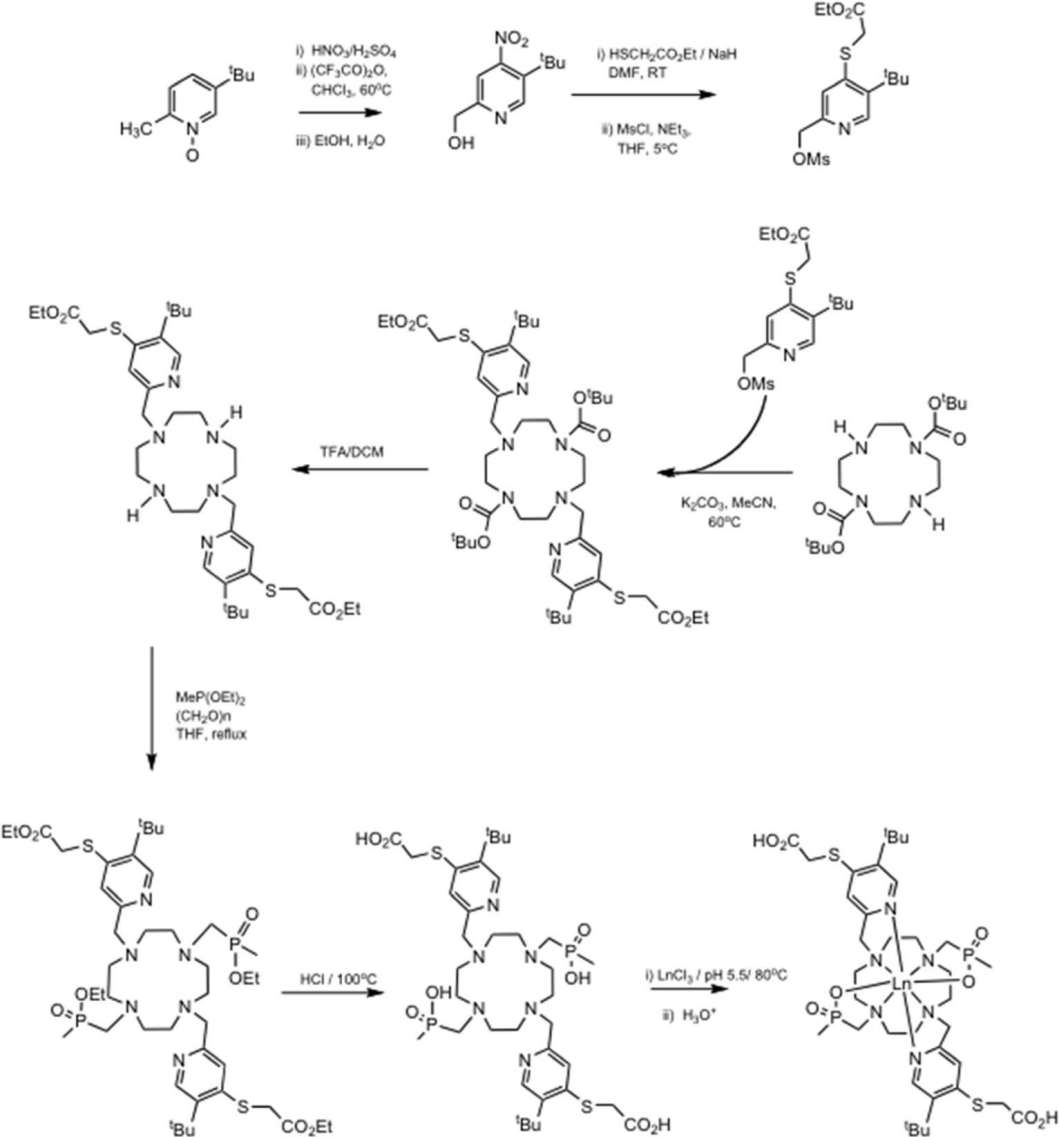
Synthesis scheme for the [Ln.L^1^]^−^ complexes, in which Ln was either Gd or Dy.

A cyclen‐based dysprosium(III) complex ([Dy.L^1^]^−^) and its gadolinium analog ([Gd.L^1^]^−^) were designed by focusing on a high number of reporter protons and their relaxation rates as the main design criteria. The complex incorporates two *tert*‐butyl (t‐Bu) reporter groups, giving 18 equivalent protons whose resonance signal was to be detected directly. The synthesis and characterization of [Dy.L^1^]^−^ and [Gd.L^1^]^−^ were undertaken using established methodology (Fig. 1/Supporting Information), as described in recent work 23, 24. The longitudinal relaxation rate of the reporter group depends on the nature of the local field experienced by those protons and varies with lanthanide ion, whereas the presence of a nonspherical electronic distribution produces the dipolar paramagnetic shift, with a magnitude dependent on the distance and angle of the reporting nuclei to the paramagnetic center. Our criteria were that *R*_1_ must be high to allow fast pulsing, and the paramagnetic shift must be sufficient to allow the shifted reporter resonance to be excited and detected separately to the tissue signals from water and fat. The shift must therefore be greater than the imaging readout bandwidth, so that there is no contamination of the PARASHIFT image by residual water or fat signal. Based on previous work, Dy was found to have more favorable relaxation properties than Tm, Tb, or Ho 23, 24; therefore, [Dy.L^1^]^−^ was chosen for PARASHIFT imaging, whereas [Gd.L^1^]^−^ was synthesized for standard MR contrast imaging.

High‐resolution NMR field‐dependent *R*_1_ measurements were made, examining the *tert*‐butyl resonance. The nuclear relaxation times of the *tert*‐butyl group of [Dy.L^1^]^−^ were measured at six field strengths (4.7, 9.4, 11.7, 14.1, and 16.5 Tesla (T)) at 295 K using the inversion‐recovery technique (see Supporting Information). The nuclear relaxation data were fitted by using a modified MATLAB algorithm (The MathWorks, Natick, Massachusetts) originally written by Dr. Ilya Kuprov (Southampton University). The algorithm uses the Solomon‐Morgan‐Bloembergen equation (Eq. (1)) to fit the measured relaxation data using Levenberg‐Marquardt minimization of the nonlinear squares error function, from which the electron‐nuclear distance *r* was estimated:

(1) $$R_{1}=\frac{2}{15}{\left(\frac{\mu_{0}}{4\pi }\right)}^{2}\frac{\gamma_{N}^{2}\;g_{\text{Ln}}^{2}\;\mu_{B}^{2}\;J\left(J+1\right)}{r^{6}}\left[\frac{3T_{1e}}{1+\omega_{N}^{2}T_{1e}^{2}}+\frac{7T_{1e}}{1+\omega_{e}^{2}T_{1e}^{2}}\right]+\frac{2}{5}{\left(\frac{\mu_{0}}{4\pi }\right)}^{2}\frac{\omega_{N}^{2}\;\mu_{\text{eff}}^{4}}{{\left(3k_{B}T\right)}^{2}\;r^{6}}\left[\frac{3\tau_{r}}{1+\omega_{N}^{2}\tau_{r}^{2}}\right]\;$$

where μ_0_ is the vacuum permeability, γ_N_ is the nuclear gyromagneitc ratio, g_Ln_ is the Landé factor of the Ln ion, μ_B_ is the Bohr magneton, τ_r_ is the rotational correlation time, ω_N_ is the nuclear Larmor frequency, ω_e_ is the electron Larmor frequency, and μ_eff_ is the effective magnetic moment. The results were analyzed iteratively assuming that longitudinal and transverse electronic relaxation times (*T*_1e_ and *T*_2e_) were of similar magnitude.

### MRI Studies—General Details

In vivo imaging was performed using a 7T preclinical MRI system (20‐cm bore, Varian Direct Drive scanner, Agilent, Palo Alta, California) equipped with a 39‐mm i.d. quadrature birdcage RF coil (Rapid Biomedical GmbH, Germany) for excitation and detection of the water and PARASHIFT agent signals. Mice were mounted in a dedicated bed, which included a pneumatic pillow system to measure the gate acquisition to animal respiration and a fiber‐optic thermometry system for temperature monitoring and control using a warm air system (SA Instruments, Stony Brook, New York). Mice were anaesthetized using isoflurane, and an intravenous line was inserted into a tail vein to allow injection of contrast agent from outside of the magnet. No other surgical preparation was used. To confirm positioning and visualize regional anatomy, conventional spin‐echo MRI scans were collected on the water resonance (pulse repetition time (TR)/echo time (TE) = 2200 (gated)/10.93 ms, 45 × 1 mm thick slices, field of view (FOV) 35 × 35mm, matrix 256 × 256). All animal experiments were performed in compliance with the UK Government Home Office under the Animals (Scientific Procedure) Act 1986.

### Gd Uptake and Biodistribution Study

Biodistribution of the complexes was first studied using the Gd analog in four HCT116 tumor‐bearing CD1 Nu/Nu Nude mice (Charles River, UK), although the analysis only focused on normal tissues. Tissue uptake and clearance of the complex was examined by dynamic contrast enhanced (DCE) MRI using [Gd.L^1^]^−^, providing T_1_w contrast on conventional MRI. Slices were chosen through the liver and kidneys. Gradient echo scans were acquired every 6 s for 60 min, commencing five images before administration of the contrast agent (dose of 0.1 mmol/kg [Gd.L^1^]^−^, in a volume of 200 µl given over 6 s). MRI parameters were TR/TE = 23.45/4.40 ms; flip angle 30°, 2‐mm slice thickness, FOV 40 × 40 mm, matrix 256 × 256. DCE images were analyzed using ImageJ software (http://imagej.nih.gov/ij/) to extract image intensity changes over time. Regions of interest (ROI) were drawn on liver, kidney, muscle and bladder, and mean ROI intensity measurements were calculated for each time point. Data were normalized to the mean background level before contrast injection.

Following MRI, mice were sacrificed by cervical dislocation and tissues excised immediately and freeze‐clamped in liquid nitrogen. To obtain tissue concentrations over time, a further series of animals (*n* = 3 per time point) were injected with [Gd.L^1^]^−^ and sacrificed at 10, 20, 40, and 80 min post injection. In all cases, three separate tissue samples were taken from the kidney and liver and stored at −80°C before the analysis. Plasma samples were taken at each time point and frozen. Samples were analyzed for Gd content using ICP‐mass spectrometry (see Supporting Information).

### PARASHIFT Dynamic Imaging Studies

PARASHIFT studies using the [Dy.L^1^]^−^ were performed in CD1 wild type mice (Charles River, UK) without any implanted tumor (*n* = 6). Unlike previous in vivo studies using intravenous injection of paramagnetically shifted agents 8, 9, 14, 15, 16, 17, 18, 25, our animals were physiologically intact, without renal ligation. Animals were positioned in the birdcage coil and a 20‐mm long, 5‐mm diameter NMR tube phantom containing ∼100 μL of 6‐mM solution of PARASHIFT agent positioned under the body of the animal to allow for system calibration (scanner frequency) and to act as an external reference for quantitation. This phantom was fastened to a line that allowed it to be withdrawn from the imaging FOV following calibration to avoid contamination of the in vivo signal from the phantom. Measurements were made in three groups of animals using three different methods to illustrate different aspects of the PARASHIFT molecular signal.

### Measurement 1—Optimized Imaging of Regional Contrast Distribution

For simple detection of the agent in vivo, an optimized 3D gradient‐echo (3DGE) sequence was employed. Our previous work has shown that once an imaging spectral width (SW) is defined (which, with the choice of imaging matrix size determines the minimum TE), the scan should be collected at the shortest available TR under Ernst angle conditions 21. The minimum allowed SW for these experiments is defined by the fat‐PARASHIFT frequency difference. At the 7T operating frequency of 300 MHz, this frequency difference (1.5–61 ppm) corresponds to ∼20 kHz. By restricting the imaging bandwidth to 20 kHz and applying a sharp digital filter to the data, images of the water or PARASHIFT peak were collected simply by shifting the excitation and acquisition center frequency of the scan. Signal excitation used a nonspatially selective 1‐ms‐duration Gaussian excitation pulse with a FWHM bandwidth of 2100 Hz, falling to a fractional excitation of <10^−3^ at 20 kHz, which completely eliminated any observable contaminating signal from one resonance while imaging the other. Scan parameters were as follows: TE/TR = 1.45/2.87 ms (ungated), 20‐kHz spectral width, axial FOV 64 × 64 mm, matrix 32 × 32, 16 encodes in the third direction covering a slab thickness of 240 mm, yielding 15‐mm thick slices. Scans used 42 signal averages for a total duration of 61.7 s per data set. Pulse flip angle was calibrated to the Ernst angle of 46°, which is possible for these experiments because the PARASHIFT *R*_1_ is known and fixed by the intramolecular interaction with the Ln ion, independent of tissue concentration. Dual detection of nonoverlapping water and PARASHIFT images were also collected using signal excitation at the PARASHIFT frequency with double receiver bandwidth of 40 kHz centered between the water and PARASHIFT frequencies and double the number of readout matrix points.

The radiofrequency (RF) excitation power was calibrated on the water signal, and then the system was retuned to the PARASHIFT frequency using the phantom signal. Imaging FOV was positioned based on standard proton MRI and the 3DGE sequence collected. This scan provided intensity reference data for the PARASHIFT phantom from which in vivo studies were quantified. The phantom was then withdrawn from the FOV and a repeat 3DGE sequence was collected to ensure that the image matrix was free of any signal from tissue water. Dynamic time series of 3DGE scans were then collected, commencing with the injection of 200 μl of PARASHIFT agent (0.04 mmol/kg [Dy.L^1^]^−^), followed by saline flush and continuing for 30 min. Following PARASHIFT imaging, the system was retuned to the water frequency and a high‐quality conventional MRI scan was collected as an anatomical reference image using a standard multislice SE sequence (respiratory gated TR/TE = 2200/10.9 ms, 50 × 1 mm thick slices, FOV 35 × 35 mm, matrix 256 × 256).

PARASHIFT images were analyzed using ImageJ software to extract image intensity changes over time. ROIs were drawn on tissues of interest (liver, kidney, and bladder), and mean ROI intensity values were extracted for each time point. The preinjection data set containing the PARASHIFT‐filled phantom was used as the concentration standardization reference level. The mean signal from pixels placed centrally within the phantom (completely filled with solution) was determined and PARASHIFT tissue concentration curves were calculated as the ratio of the ROI mean signal relative to the phantom, scaled by phantom concentration (6 mM). A ROI placed outside the animal was used to measure the background noise floor in the scans, to estimate the detection limit of the contrast agent.

### Measurement 2—Spectroscopic Imaging

To demonstrate detection of the signal spectroscopically rather than by imaging, two‐dimensional (2D) and 3D spectroscopic imaging sequences were used. Sequence parameters for 2DSI were TR/TE = 4.46/0.70 ms, FOV 64 × 64 mm, matrix 16 × 16, spectral sweep width 20 kHz, 64 data points. Scans used 53 signal averages with a scan duration of 61 s. The resulting data was three‐dimensional (two spatial, one spectroscopic) with the spatial dimension localizing a thick axial (XY) plane, condensing all detail along the *z*‐direction of the magnet. Sequence parameters for 3DSI were TR/TE = 7.69/0.73 ms, FOV 32 × 32×64 mm, matrix 16 × 16×8, resulting in 8‐mm thick axial slices. Spectral sweep width was 20 kHz with 128 data points. Scans used four signal averages with a scan duration of 63 s. For the 3D sequence, the resulting data was four‐dimensional (three spatial, one spectroscopic). In both cases, the signal excitation once again used the 1‐ms nonselective Gaussian pulse as in the 3DGE imaging studies.

### Measurement 3—Temperature Mapping Studies

The chemical shift of the PARASHIFT signal is sensitive to sample temperature, and can therefore be used for temperature mapping. To determine the temperature coefficient, in vitro measurements of the frequency shift against temperature were made for the [Dy.L^1^]^−^ complex in both deuterated water and rat plasma using standard temperature controlled high‐resolution NMR over the range 298–318 K, spanning room to body temperature. As pH can influence chemical shift, measurements were also made while varying the pH between 4.6 and 7.6.

In vitro temperature mapping studies were made using a sample of [Dy.L^1^]^−^ in a 5‐mm NMR tube, which was warmed in a water bath to 40°C and then placed in a polystyrene thermal insulating sleeve and imaged every 60 s as it cooled to room temperature. Sample temperature was monitored with the in vivo thermometry system. Measurements were made using a 2DSI sequence with TR/TE = 27.05/0.7 ms, spectral width 20 kHz, 512 sample points, 90° Gaussian excitation, FOV 32 × 32 mm, matrix 32 × 32, and a single average for an acquisition time of 27 s. The 2DSI data were processed using 3D Fourier transformation, including 75‐Hz exponential line‐broadening in the spectral domain. The frequency of the major PARASHIFT line was determined at each temperature by peak peaking (the presence of the smaller second isomer resonance does not confound this spectroscopic measurement). To ensure frequency shifts were only caused by temperature and not by differences in local B_o_ field strength, a separate reference scan was collected at the water frequency. Data were also presented as images displaying signal intensity at a specific spectral frequency.

In vivo temperature mapping studies were performed in CD1 mice using the same experimental protocol as the imaging experiments, but with the spectroscopic imaging sequence. The in vivo measurements used a 3DSI sequence with TR/TE = 7.69/0.70 ms, spectral width 20 kHz, 128 sample points, 90° Gaussian excitation, FOV 32 × 32 × 64 mm, matrix 16 × 16 × 8, and four averages for a total acquisition time of 63 s per data set. Measurements were made preinjection and at 1, 2, 3, 4, 5, and 25 min post injection. The 3D spectroscopic imaging data were processed using four‐dimensional Fourier transformation, including 75‐Hz exponential line‐broadening in the spectral domain. Maps of local tissue concentration of agent were created by integration of peak area for every pixel in the 3D spatial volume. Regional temperature differences were determined from peak frequency changes.

## RESULTS

**Figure 2:**
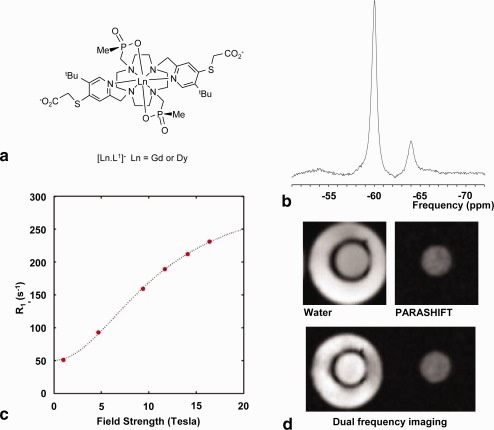
Structure and properties of the PARASHIFT complex. **(a**) Structure of [Ln.L^1^]^−^. (b) Proton spectrum collected on a 7T preclinical imaging scanner from the *tert*‐butyl signal region (centered at −60.1 ppm) for [Dy.L^1^]^−^. The signal was measured from 100 μL of 6 mM solution using a volume imaging coil, with a 1‐ms‐long Gaussian 90° excitation pulse, 20‐kHz spectral width, TR = 55 ms, 32 averages, and a total acquisition time of 1.76 s. The long RF pulse was used to narrow the bandwidth and prevented excitation of water, but led to a first‐order phase difference between the major and minor resonances. The major resonance at −60.1 ppm yields 88% of the signal, with the minor resonance at −63.8 ppm the remaining 12% signal. (c) Longitudinal relaxation rates for [Dy.L^1^]^−^ as a function of magnetic field, (D_2_O 295 K) showing the fit (line) of the Solomon‐Morgan‐Bloembergen equation to the data (fixed *r* = 6.5 Å; *τ*_r_ = 334 ps; *T*_1E_ = 0.41 ps; *μ*_eff_ = 10.6 B.M.). (d) Axial images from a 3DGE acquisition in a concentric tube phantom containing 3 mM [Dy.L^1^]^−^ solution in the central tube and water only in the outer tube. Upper row of the panel shows the water (left) and [Dy.L^1^]^−^ (right) images using frequency‐selective excitation of each resonance. Lower row of panel shows dual imaging acquisition using double bandwidth readout and Gaussian signal excitation optimized at the [Dy.L^1^]^−^ frequency. Residual flip angle at the water frequency (20‐KHz offset) yields the water image.

The structure of the [Ln.L^1^] complex is shown in Figure 2a. The proton spectrum of [Dy.L^1^]^−^ (Fig. 2b) shows the t‐Bu PARASHIFT signal at −60.1 ppm (295 K, a frequency shift of ∼20,000 Hz relative to water at 7 T) and a smaller peak (12%) at −63.8 ppm. These peaks arise from two isomers that are in slow chemical exchange. The smaller peak is believed to be a stereoisomer with P centers of the same configuration (SSS) and helicity Lambda, whereas the major isomer is SSS‐Delta. Each tBu resonance relaxes at the same rate, consistent with this assignment. Related triphosphinate lanthanide(III) complexes have previously been examined, and assignments are consistent with that work 26. Signals from the PMe groups are not seen in this spectral region, as they resonate at +52, + 90, and +99 ppm, between 110 and 160 ppm away from the t‐Bu resonances. The t‐Bu group to metal ion distance was estimated at 6.5 (±0.1)Å, following previous density functional theory calculations of similar structures 24. This estimate is supported by the excellent fit between the NMR field‐dependent *R*_1_ measurements of the *tert*‐butyl group and Bloch‐Redfield‐Wangsness theory 23, 24 using a distance of 6.5Å (Fig. 2c). It is important to stress that the spectral peak arises directly from the *tert‐*butyl protons, and that the *R*_1_ relaxation rate is independent of injected concentration—a fundamental distinction from conventional MR agents, in which the water relaxation rate varies proportionally with instantaneous contrast agent concentration in the tissue. Relaxation rate *R*_1_ was measured at 7 T to be 128 s^−1^, with *R*_2_ of 227 s^−1^ (*R*_1_/*R*_2_ = 0.56). Figure 2d shows phantom images illustrating the 3DGE images from the individual water or PARASHIFT resonances, and using simultaneous dual PARASHIFT‐water acquisition.

**Figure 3:**
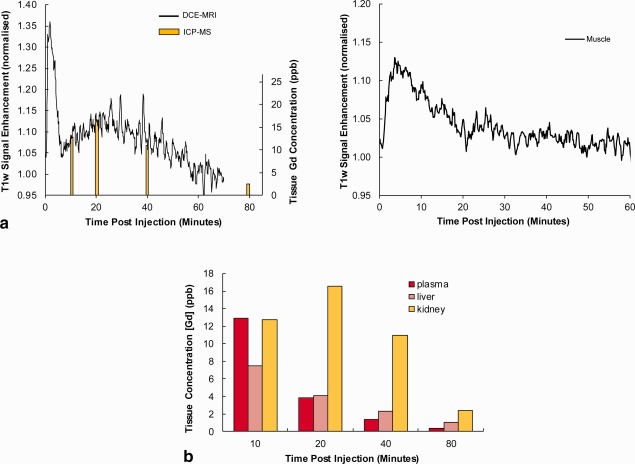
Biodynamics of [Gd.L^1^]^−^ in vivo. (a) MRI signal intensity curves obtained from selected ROIs using a DCE‐MRI sequence. (b) Measurements of tissue Gd concentration based on invasive tissue sampling. The kidney data (yellow) are shown overlaid onto the DCE‐MRI signal curve (a, left) to illustrate the similarity in time course (despite being in different animals).

The biokinetics study of the complex using [Gd.L^1^]^−^ and DCE‐MRI (conventional, indirect *T*_1_ contrast) is illustrated in Figure 3. DCE‐MRI data demonstrated a biphasic kinetic profile in the kidney with an early peak ∼2 min, arising primarily from the intravascular signal of the injected agent (Fig. 3a), followed by a second broader peak, as the complex exchanged in and out of body tissues and was cleared through the kidneys. In muscle, the tissue contrast peaked at ∼5 min and then decayed away over the ensuing 40 min, with the [Gd.L^1^]^−^ complex becoming visible in the bladder and continually increasing over the following 45 min (data not shown). Tissue Gd concentration was separately analyzed by inductively coupled plasma mass spectroscopy of excised tissue, and strongly paralleled the MRI data, as expected (Fig. 3).

**Figure 4:**
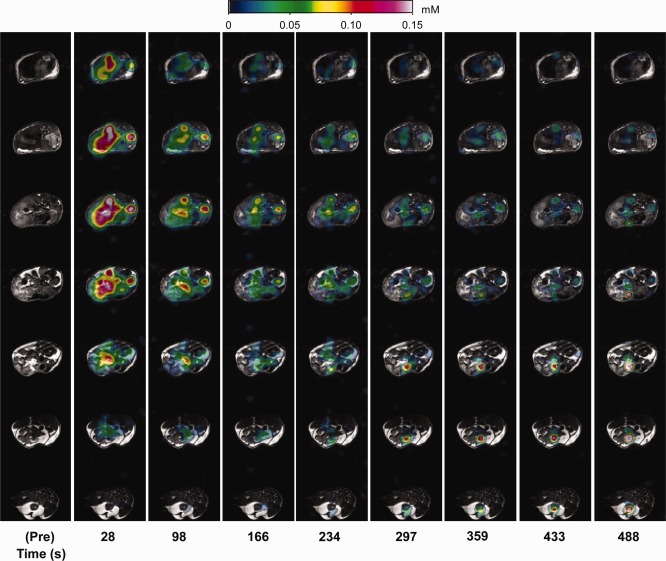
PARASHIFT measurements in vivo. PARASHIFT signal from [Dy.L^1^]^−^ (color scale) overlaid onto conventional structural MRI scans. Each column represents a different time point post injection. Within each column the data represent different spatial axial slices through the mouse. Mean peak ROI signal to noise ratio in this animal was 9.9 in liver, 11.7 in kidney, and 18.6 in bladder.

**Figure 5:**
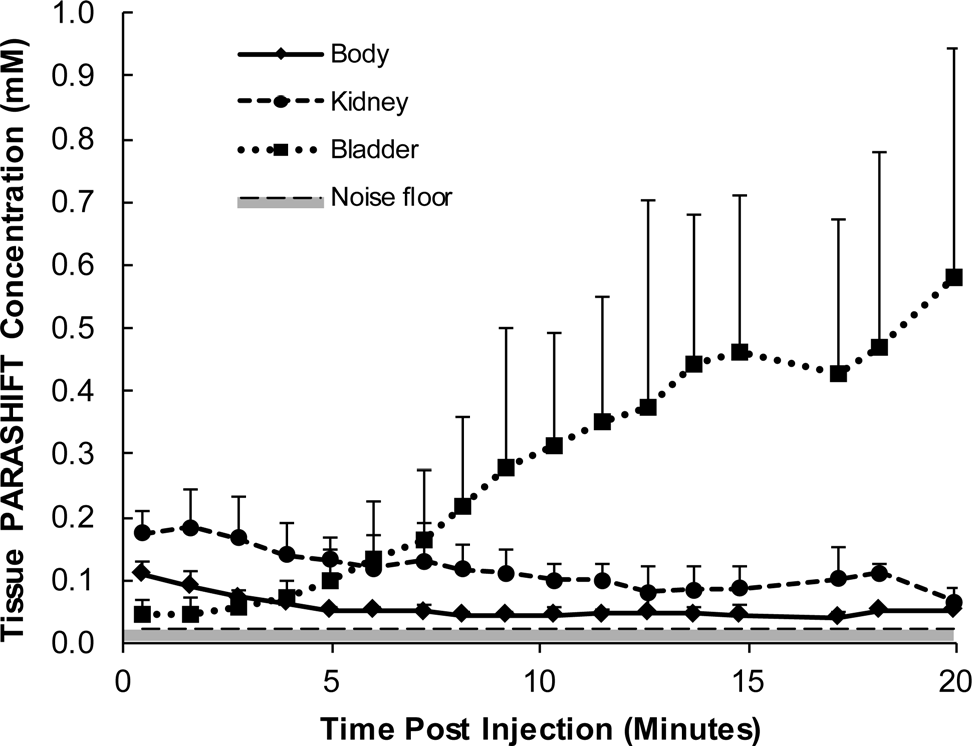
Time series analysis of PARASHIFT concentration from selected ROIs in six mice.

Figure 4 illustrates the PARASHIFT molecular MRI measurements (direct detection of the *tert‐*butyl group of the [Dy.L^1^]^−^ complex). Passage of the agent through the tissue could be followed by 3D imaging (Fig. 4), and ROI analysis demonstrated similar biokinetics to the Gd analog (Fig. 5). Mean peak SNR was 14.8 (range 8.9 to 22.1) in kidney, 7.2 (range 4.0 to 10.0) in liver, and 30.0 (range 12.7 to 45.5) in bladder. Because the signal in the PARASHIFT images arises from the molecule itself and the *R*_1_ relaxation rate is intrinsic to the intramolecular interaction, the signal was quantified against the external reference solution acquired under the same conditions. Peak concentration of PARASHIFT measured in the kidney and liver ROIs was determined to be 200 ± 90 and 90 ± 20 μmol dm^−3^, respectively. Under these conditions, the detection limit, as determined by the noise floor in the scans, was estimated to be 23 μmol dm^−3^.

**Figure 6:**
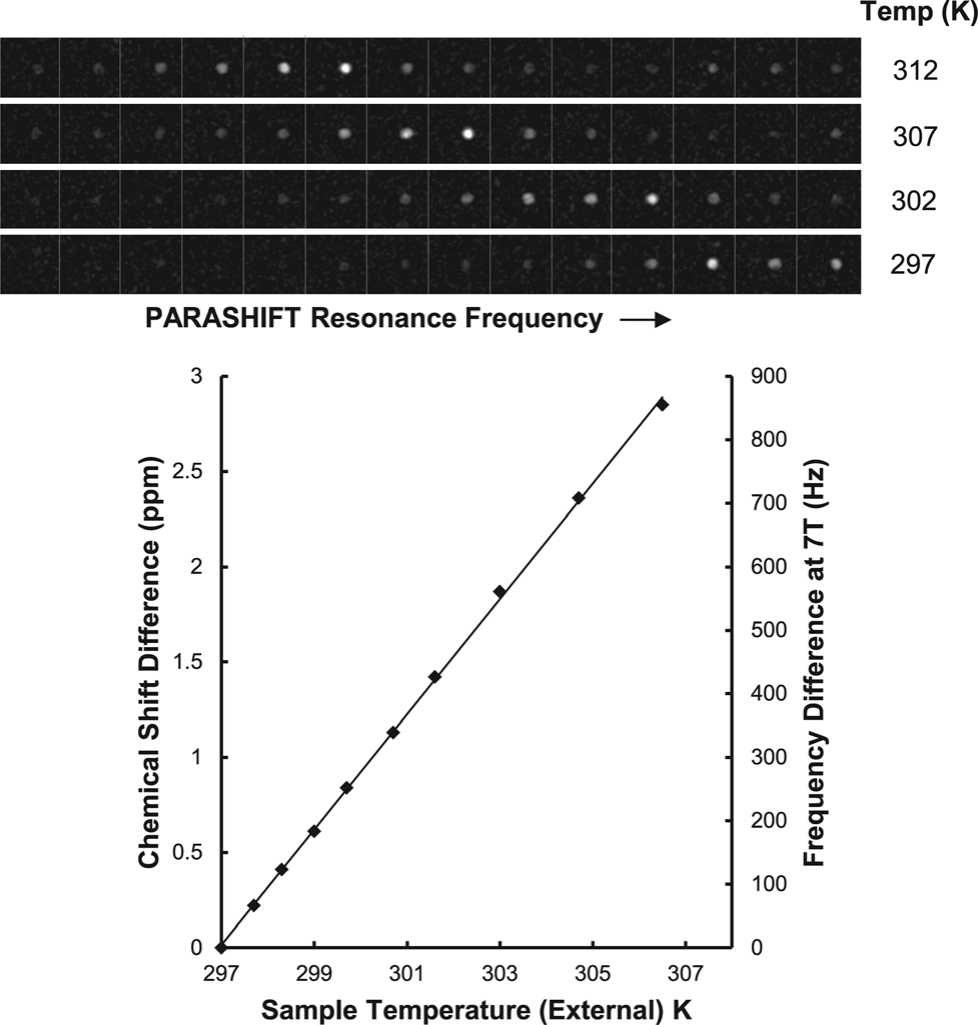
In vitro PARASHIFT temperature mapping studies. *E*ach image row presents data collected using a 2D spectroscopic imaging acquisition at the specified sample temperature. Chemical shift separation between each image is 0.52 ppm. The spectral data were reconstructed as images of PARASHIFT peak intensity at each spectral frequency, and dependence of shift on temperature is plotted. The upper image rows (T = 307 K and 312 K) span both the major and minor isomers and demonstrate that these peaks shift in parallel; therefore, the presence of the minor isomer does not confound temperature measurement using the major isomer.

**Figure 7:**
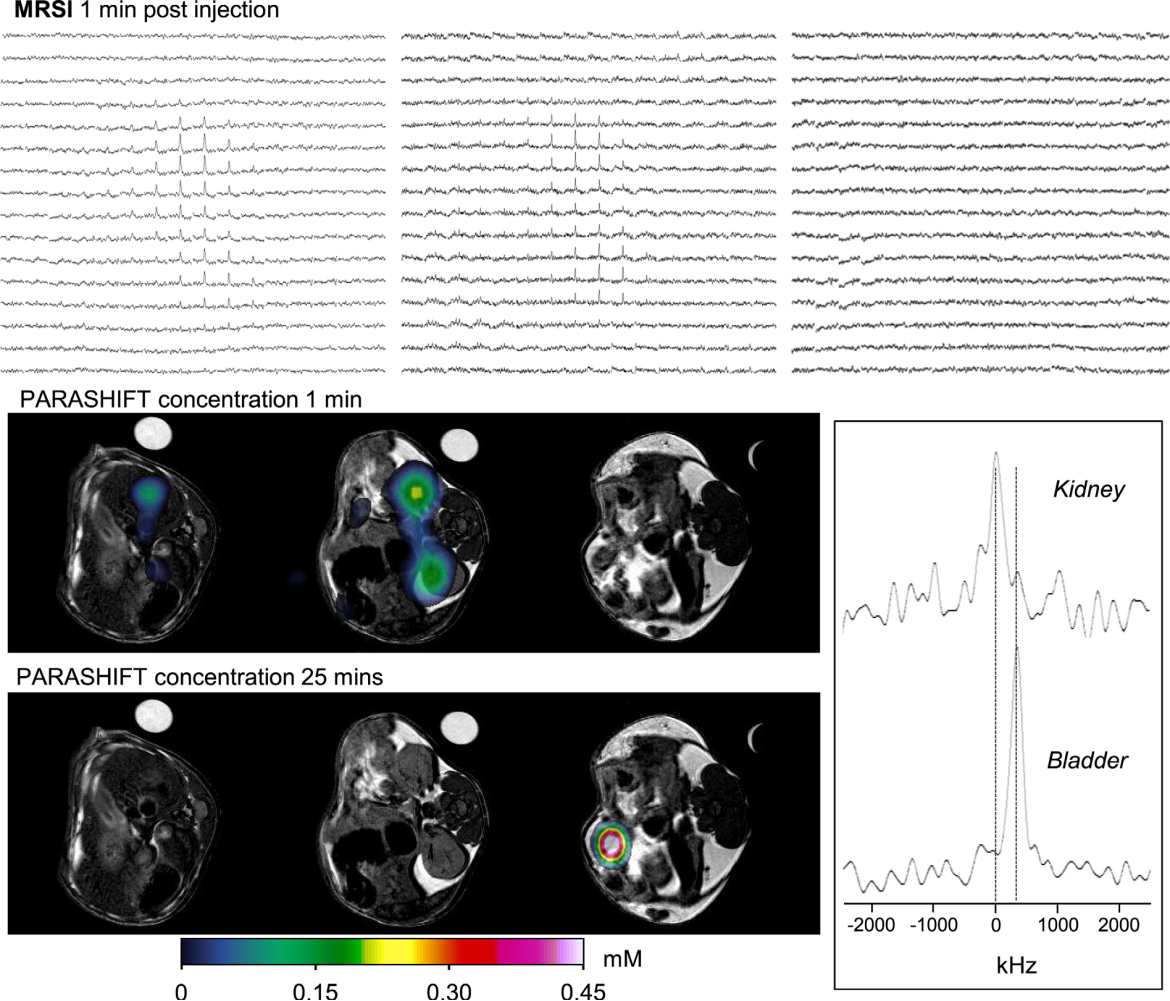
In vivo PARASHIFT dual imaging experiment showing contrast agent distribution as a function of time and tissue temperature assessment based on the frequency dependence of the PARASHIFT signal. Data were collected using a 3DSI sequence providing a four‐dimensional data set (three spatial and one spectral). The image panel presents the spectral grids for three of the MRSI slices acquired 1 min after intravenous injection (upper row), with the same data displayed as the reconstructed PARASHIFT tissue distribution (derived from the peak area for each voxel in the 3DSI experiment) overlaid on the anatomical scans (middle row). The tissue concentration data at 25 min post injection are shown in the bottom row. The anatomical scans were collected before contrast injection and show the location of the PARASHIFT filled tube used for system calibration and as a concentration reference. This sample tube was withdrawn remotely from the FOV before injection and therefore does not appear in the PARASHIFT images. PARASHIFT frequency is temperature‐dependent and can be used to map temperature differences. Spectra (lower right panel) were extracted from selected regions of interest in kidney at the 1‐min time point and from the bladder at the 25‐min time point and corrected for differences in B_o_ field strength in each region based on the water signal frequency. Significant changes in signal frequency (temperature) are apparent over time between the kidney and bladder.

The relationship between PARASHIFT frequency and sample temperature was determined in vitro to be linear over the range 298–318 K (Supp. Figs. S1–S3 and Fig. 6) with a temperature coefficient of 0.25 ± 0.03 ppm/K in D_2_O and 0.28 ± 0.01 ppm/K in mouse plasma. At the 7T field used for imaging, this corresponds to 84 Hz/K, which is small enough not to cause spatial distortion in the image formation process (image bandwidth 312.5 Hz per pixel), but is sufficiently large to allow accurate measurement by optimized MRSI. Temperature mapping data from the in vitro study is illustrated in Figure 6, which shows spectroscopic images reconstructed across the linewidth of the main PARASHIFT peak. Signal from the minor isomer is seen to the right and shifts in parallel with the major isomer. Measurement of chemical shift as a function of pH revealed that frequency was unchanged between pH of 4.6 and 7.0 and then changed by 0.1 ppm at pH 7.6. Spectroscopic imaging allowed the PARASHIFT peak frequency to be measured in vivo and converted to tissue temperature (Fig. 7) based on the measured temperature coefficient. Although the animal's core body temperature was maintained at 37°C, the shift‐mapping studies demonstrated temperature variations between tissues over time, which decreased by 348 Hz (4.1 K) between initial detection of the intravascular signal and arriving in the bladder.

## DISCUSSION

MRI is an inherently insensitive detection method, and the ability to obtain direct molecular images by MRI under thermal equilibrium magnetization conditions is limited to compounds with tissue concentrations typically in the low millimolar range. Approaches to enhance the sensitivity to molecular targets have used conjugation of the target molecule to large reporter compounds such as iron oxide particles, which yield contrast via indirect (and often diffuse) through‐space interactions, or other intermolecular interactions via chemical exchange, such as in CEST and PARACEST 22 methods. The use of paramagnetically shifted resonances arising from nuclei within the structure of the target molecule 8, 9, 15, 17, 18, 19, 23 offers an alternative method based on small molecule structures, but optimization of the chelator structure to maximize imaging sensitivity has seen little systematic investigation.

Relaxation rates are a key factor in defining detection sensitivity. By manipulating the intramolecular distances from the lanthanide ion to the reporter group, the *R*_1_ relaxation rate can be increased, allowing the MR sequence to be run rapidly and with high flip angle, maximizing the signal collection per unit time 21. *R*_1_ for the [Dy.L_1_]^−^ probe was 128s^−1^ at 7 T, rising to 160 s^−1^ at 9.4 T and 185 s^−1^ at 11.7 T, which is slightly lower than the relaxation rate of the H‐3 protons in [TmDOTMA] (reported to be 188 s^−1^ at 9.4 T 9 and 211 s^−1^ at 11.7 T) 16 and substantially lower than for the H‐6 protons of TmDOTP (reported as 625 s^−1^ at 11.7 T) 16. Although this might suggest TmDOTP has preferred properties, sensitivity is not solely defined by *R*_1_, but also by transverse signal decay (*R*_2_), which reduces the overall signal. The very high reported *R*_2_ for TmDOTP at 11.7 T (1369 s^−1^) 16 indicates that transverse signal loss is significant for imaging, and linewidths are extremely broad for spectroscopic detection. *R*_1_/*R*_2_ for TmDOTMA are reported as 0.883 at 11.7 T 16 and 0.774 at 9.4 T 9. However, excretion is very fast and the structure presents no opportunity for structural modification, as the equivalence of the 4 Me groups or 4 H on the ring is lost by anything other than C‐4 symmetric tetra‐substitution.

Choice of the lanthanide metal is fundamental to the magnitude of the paramagnetic shift, as has been illustrated here for dysprosium (60‐ppm shift) and gadolinium (no shift). Selection of other lanthanide ions, such as Tb, Tm or Er, provide agents with different properties both in terms of reporter group relaxation rates and paramagnetic shift. The more commonly studied thulium complexes show paramagnetic shifts from 140 ppm 11 to > 200 ppm 9, whereas characterization of similar Ln structures to that used here shows that shifts ranging from approximately −80 to + 70 ppm are achievable 24. Our recent work suggests that these shifts are not entirely predicted by current theory 27, but the experimentally demonstrated shifts offer opportunities for more advanced imaging approaches.

Previous work observing paramagnetically shifted ligand resonances has been limited by the need for large doses, almost always requiring the use of renally ligated animals to eliminate clearance of the agent, or the use of continuous infusion to maintain sufficient signal intensity. For example, the use of the rapidly cleared complexes, [Tm.DOTP]^5‐^ or [Tm.DOTMA]^−^, has been examined 8, 14, in which the temperature and pH dependence of a shifted resonance was monitored by spectral imaging in rats undergoing continuous infusion to maintain a complex concentration in the blood of the order of 2–3 mmol/kg. In the current work we successfully imaged the dynamics of the complex using a single intravenous injection in an intact animal at a dose of 0.04 mmol/kg of [Dy.L^1^]^−^. Previous studies have also achieved high spatial resolution of 1‐μl voxels 14, but again this has only been possible using high administered doses in combination with small surface coils, leading to restricted FOV. In studying dynamics, our focus was on temporal rather than spatial resolution; therefore, we selected a lower spatial resolution. However, taking into account differences in B_o_ field strength, scan duration, total complex administered, and the sensitivity differences between surface coils and our volume coil 28, we calculate that our data show a sensitivity improvement of a factor of 5 over this high resolution study 14, and factors of 20 18 and 60 9, 16, 17 against other recent studies using [TmDOTMA]^−^. These numbers are conservative estimates, as they make no allowance for our use of a single intravenous injection. More recently, [Tm.DOTMA]^−^ has been used in cell‐labelling studies in vivo, in which the complex was internalized inside the cell population before injection 19, which highlights an area for further evaluation of our complex.

We have demonstrated the ability to measure our agent in vivo at a low tissue concentration of 23 μmol dm^−3^, similar to the detection threshold for a fluorine‐based compound that we have previously reported 21. Further increases in sensitivity require higher signal level per molecule and slower clearance. Increasing the signal is possible in imaging experiments through the use of partial Fourier acquisition or ultrashort TE (UTE) imaging 19, to reduce signal loss from transverse relaxation. In theory, a factor of ∼1.3 is achievable from the relaxation loss during the current 1.45‐ms TE and the *R*_2_ of our molecule (227 s^−1^). The rate of clearance and excretion of the contrast agent is also relatively high. Altering structure, such as conjugation of multiple copies to a larger molecule 29, can be used to change biodynamics and further enhance detection sensitivity.

We have demonstrated temperature mapping using this agent as a simple physiological probe, although we acknowledge temperature can be measured using conventional water MRI. Although the in vitro data demonstrate the strong temperature dependence of the chemical shift, other factors may affect the signal frequency, including local B_o_ variation and tissue pH. Because B_o_ inhomogeneity affects the PARASHIFT and water signal frequencies equally, the in vivo PARASHIFT data were corrected for inhomogeneity by referencing the water frequency measured in each pixel using the MRSI sequence tuned to the water resonance. Further in vitro measurement demonstrated that shift was essentially insensitive to pH. Therefore, while our data would be strengthened by an independent in vivo temperature reference, we conclude that the main source of chemical shift variation is indeed temperature.

Future developments will modify the molecule to place a substituent next to the t‐butyl group whose shift is sensitive to pH, creating a pH probe. Co‐injection of PARASHIFT agents with different functional properties (eg, sensitivity to pH, T) and with different Ln ions selected to produce opposing shifts into the upfield and downfield regions of the spectrum, can then allow monitoring of multiple processes in vivo, simply by selection of the appropriate bandwidth and resonant frequency. A further possibility is to consider these types of structures as “building blocks” for more complex forms. Alternatively, linking structures based around only one species of Ln metal but with different paramagnetic shifts engineered via different Ln ion, reporter group distances could produce multifunctional probes. An example would be to create dual‐probes with distinguishable signals that are physiologically sensitive and insensitive to provide an internally referenced scan intensity. Dual or multiprobe systems would also have the advantage that biodynamics for each group would be guaranteed to be identical.

An important alternate strategy for molecular MRI employs chemical exchange effects (eg, chemical exchange saturation transfer (CEST) imaging). This approach is again an indirect detection method measuring changes in the bulk (tissue) water signal as a result of saturation of a frequency‐shifted proton that is undergoing chemical exchange between the molecular tracer of interest and the bulk water. The frequency shift may be the result of natural chemical shift effects 30 or the result of induced shifts from injected paramagnetic ions (PARACEST approaches 22, 31). For PARACEST agents, the indirect nature of the detection continues to be a challenge when determining the absolute concentration for many cases of molecule. Although a number of agents have been synthesized and evaluated for sensitivity to physiological parameters in vitro (reviewed in 32), relatively few have been successfully demonstrated in vivo 33, 34, 35, 36, 37, 38, 39, and in some cases have used direct injection into tissue of interest and/or high injected doses to obtain high local tissue concentrations. Recently, PARACEST agents have been used in mouse models to measure extracellular pH 37, 38, 39 and enzyme activity 36. In the context of the previous CEST‐based work, it is again important to stress that the PARASHIFT approach we describe here is entirely distinct and in no way relates to chemical exchange effects.

## CONCLUSIONS

This study has demonstrated a new chelator structure for high‐sensitivity molecular imaging in vivo. This agent can be detected using clinically relevant doses and used to report simultaneously on tissue concentration and physiological parameters, eg, tissue temperature.

## Supporting information


**Fig. S1.** PARASHIFT chemical shift as a function of sample temperature. **T**he chemical shift of the *tert‐*butyl group in [DyL^1^]^−^ was measured as a function of temperature in vitro, by high‐resolution NMR at 11.7 T (^1^H, 500 MHz) both in D_2_O (blue) and murine plasma (red). Linear fitting revealed a dependence of 0.31 ppm K^−1^ in D_2_O, and 0.28 ppm K^−1^ in murine plasma, in agreement with the phantom imaging study at 7 T in 0.9 *w/v* % NaCl saline solution of 0.28 ppm K^−1^.
**Fig. S2.** Chemical shift of the *tert*‐butyl resonance versus 1/T^2^ for [Dy.L^1^]^−^ (11.7 T, ^1^H) by high‐resolution NMR, over the temperature range 290–316 K.
**Fig. S3.** Longitudinal relaxation rate versus 1/T^2^ for [DyL^1^]^−^: the *tert*‐butyl resonance of approximately −60 ppm (11.7 T) was monitored by high‐resolution ^1^H NMR, over the temperature range 290–316 K.
