# Supplementary material for: A new paramagnetically shifted imaging probe for MRI
Source: Magn Reson Med. 2016 Feb 28;77(3):1307–17. doi: 10.1002/mrm.26185 (PMC5324534; doi:10.1002/mrm.26185)
Supplement: Supplementary file 1 — Fig. S1. PARASHIFT chemical shift as a function of sample temperature. The chemical shift of the tert‐butyl group in [DyL1]− was measured as a function of temperature in vitro, by high‐resolution NMR at 11.7 T (1H, 500 MHz) both in D2O (blue) and murine plasma (red). Linear fitting revealed a dependence of 0.31 ppm K−1 in D2O, and 0.28 ppm K−1 in murine plasma, in agreement with the phantom imaging study at 7 T in 0.9 w/v % NaCl saline solution of 0.28 ppm K−1. Fig. S2. Chemical shift of the tert‐butyl resonance versus 1/T2 for [Dy.L1]− (11.7 T, 1H) by high‐resolution NMR, over the temperature range 290–316 K. Fig. S3. Longitudinal relaxation rate versus 1/T2 for [DyL1]−: the tert‐butyl resonance of approximately −60 ppm (11.7 T) was monitored by high‐resolution 1H NMR, over the temperature range 290–316 K. [file MRM-77-1307-s001.docx]

**Supporting Material**

**A new paramagnetically shifted imaging probe for MRI**

P. Kanthi Senanayake^3^, Nicola J. Rogers^3^, Katie-Louise N. A. Finney^3^, Peter Harvey^3^,

Alexander M. Funk^3^, J. Ian Wilson^2^, Dara O’Hogain^1^, Ross Maxwell^2^,

David Parker^3^, Andrew M. Blamire*^1^

**Affiliations:**

1: Institute of Cellular Medicine & Newcastle MR Centre, Newcastle University

2: Northern Institute for Cancer Research, Newcastle University

3: Dept. of Chemistry, Durham University, South Road, Durham.

**Supplementary Figures**


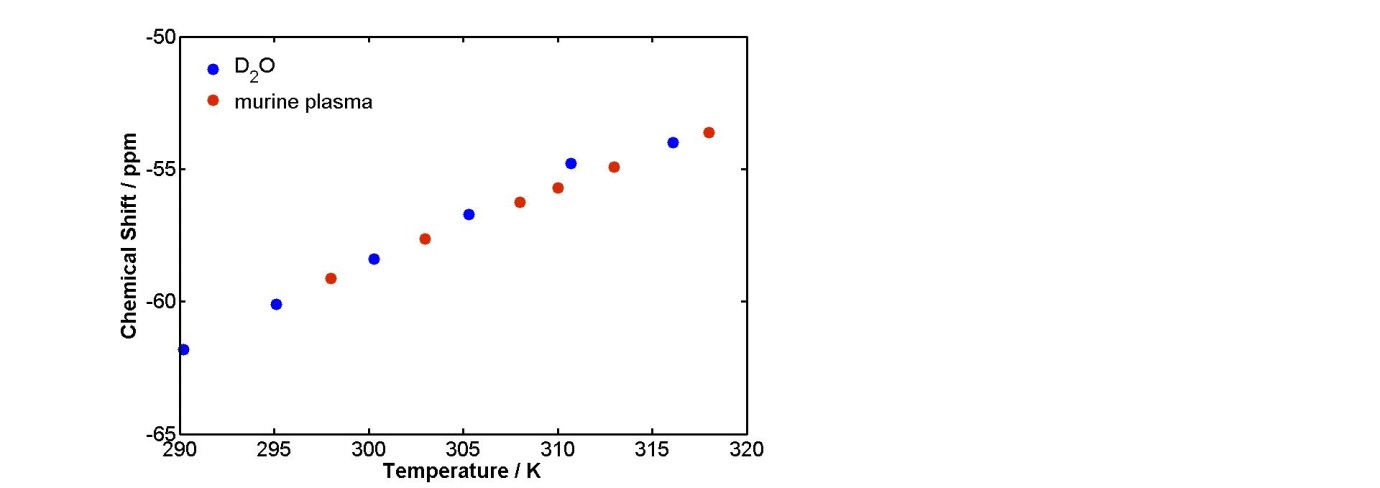


**Supporting Figure S1:** PARASHIFT chemical shift as a function of sample temperature.

The chemical shift of the *tert-*butyl group in [Dy.L^1^]^-^ was measured as a function of temperature in vitro, by high-resolution NMR at 11.7 T (^1^H ^,^ 500 MHz) both in D_2_O (*blue)* and murine plasma (*red*). Linear fitting revealed a dependence of 0.31 ppm K^-1^ in D_2_O, and 0.28 ppm K^-1^ in murine plasma, in agreement with the phantom imaging study at 7 T in 0.9 *w/v* % NaCl saline solution of 0.28 ppm K^-1^.


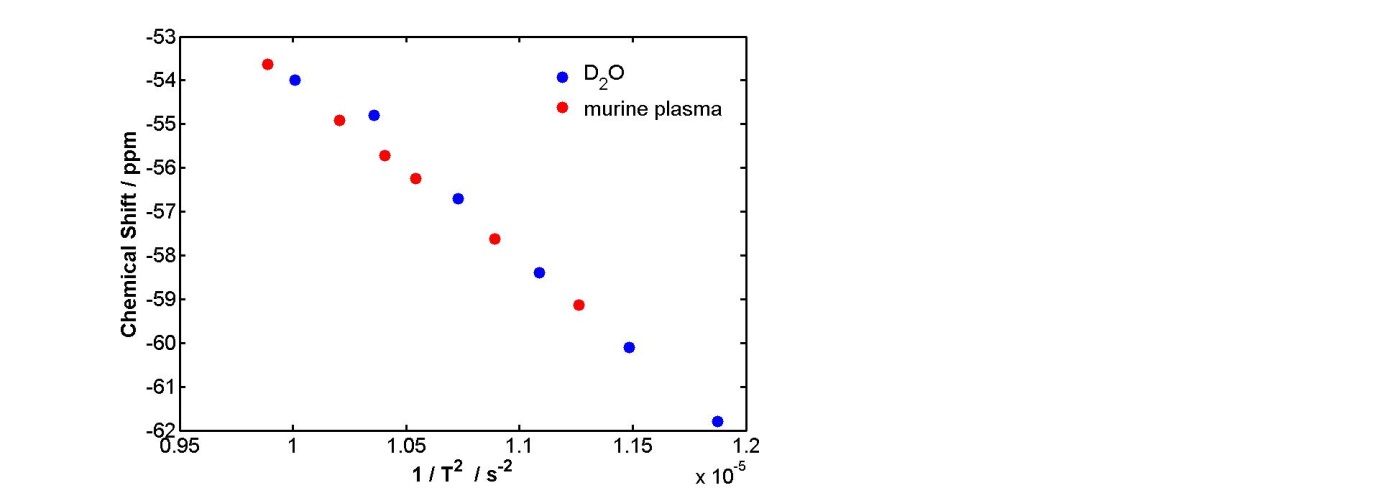


**Supporting Figure S2:** Chemical shift of the *tert*-butyl resonance vs 1 / T^2^ for [Dy.L^1^]^-^ (11.7 T , ^1^H) by high-resolution NMR, over the temperature range 290 – 316 K.


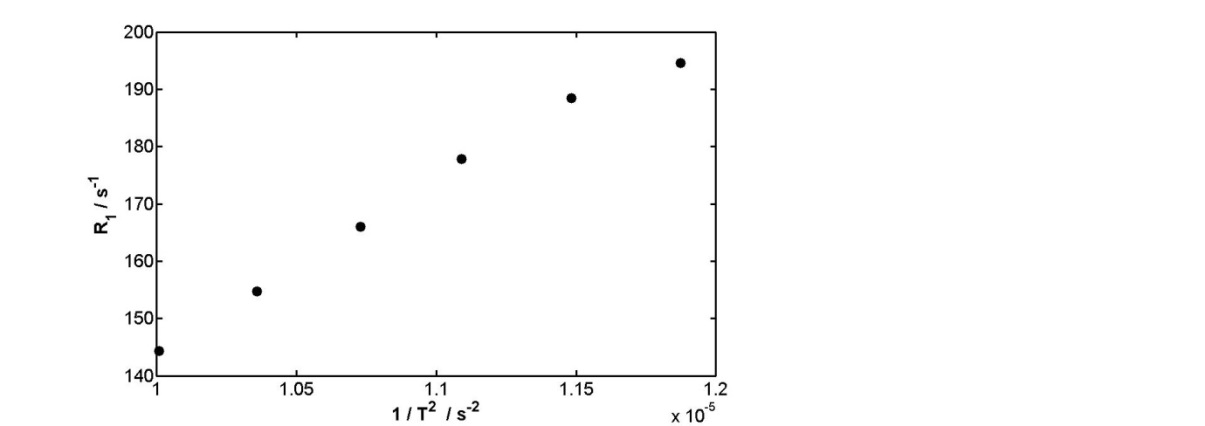


**Supporting Figure S3**: Longitudinal relaxation rate vs 1 / T^2^ for [Dy.L^1^]^-^ : the *tert*-butyl resonance around -60 ppm (11.7 T) was monitored by high-resolution ^1^H NMR, over the temperature range 290 – 316 K.

**Supplementary Methods**

*Measurement of NMR relaxation characteristics of [Dy.L^1^]^-^*

High-resolution NMR field-dependent *R_1_* measurements were made, examining the *tert*-butyl resonance of [Dy.L^1^]^-^. Proton NMR spectra were obtained at 295 K on Varian spectrometers operating at 4.7, 9.4, 11.7, 14.1 and 16.5 Tesla, specifically on a Mercury 200 spectrometer (^1^H at 200.057 MHz), a Mercury 400 spectrometer (^1^H at 399.97 MHz), a Varian Inova-500 spectrometer (^1^H at 499.78 MHz), a Varian VNMRS-600 spectrometer (^1^H at 599.944 MHz) and a Varian VNMRS-700 spectrometer (^1^H at 700.000 MHz). Commercially available deuterated solvents were used. Measurements at 1T (42.5MHz ^1^H) were made on a Magritek Spinsolve spectrometer. The operating temperature was measured using an internal calibration sample of neat ethylene glycol.

The nuclear relaxation times of the *tert*-butyl group were measured at the 6 field strengths using the inversion-recovery technique. The incremented delay time was set to span full inversion through full recovery to equilibrium of the signal. The recorded free induction decays were processed using backward linear prediction, optimal exponential weighting, zero-filling, Fourier transformation, phasing and baseline correction (by Whittaker smoothing), if necessary.

*Methods for analysis of tissue content of [Gd.L^1^]^-^*

Tissue samples (n=3 per tissue, per animal) were taken from the kidney and liver and stored at -80 ^o^C prior to analysis. Plasma samples were also taken at each time point and frozen. Weighed tissue samples (typically 10 mgs each) or a fixed aliquot of plasma (0.2 mL) were transferred into separate sample vials. Concentrated nitric acid (0.8 mL) was added and each mixture held at room temperature for a week, generating a clear pale yellow solution. The solutions were diluted to a volume of 2 mL using distilled water. Each sample was analyzed in triplicate by inductively coupled plasma mass spectrometry (ICP-MS) using a Thermo Finnigan ELEMENT light resolution select field ICP-Mass spectrometer; the mean Gd value is given; a blank sample was also run.

**Ligand and Complex synthesis**

**
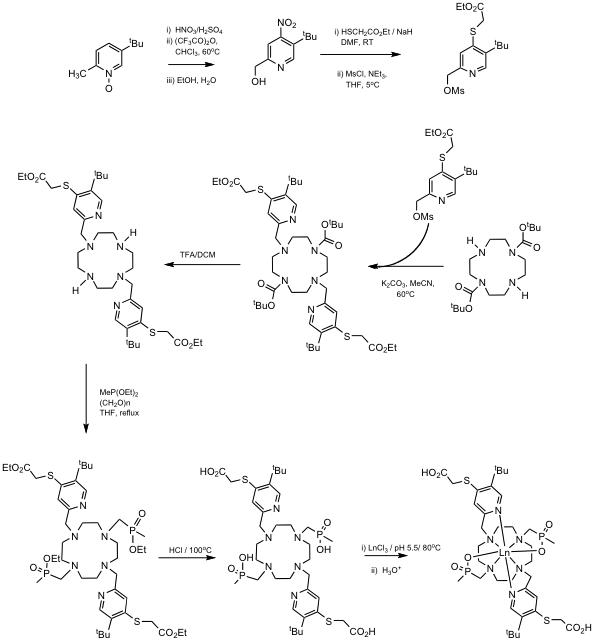
**

**HPLC**

Reverse phase HPLC was performed at 295 K using a Shimadzu system comprising a Degassing Unit (DGU-20A_5R_), a Prominence Preparative Liquid Chromatography pump (LC-20AP), a Prominence UV-Vis Detector (SPD-20A) and Communications Bus Module (CBM-20A). For preparative HPLC an XBridge C18 OBD column was used (19 x 100 mm, 5 µm) with a flow rate of 17 mL/min. For analytical HPLC a Shimadzu Shim-Pack VP-ODS column was used (4.6 x 150 mm, 5 µm) with a flow rate of 2.0 mL/min. Fraction collection was performed manually. A solvent system of H_2_O (0.1% HCOOH) / CH_3_OH (0.1% HCOOH) was used with gradient elution as follows:

| **Time / min** | **%H_2_O** | **%CH_3_OH** |
| --- | --- | --- |
| 0 | 90 | 10 |
| 3 | 90 | 10 |
| 13 | 0 | 100 |
| 16 | 0 | 100 |
| 17 | 90 | 10 |

**5-*tert*-Butyl-2-methyl -4 nitropyridine 1-oxide**

**
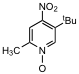
**

5-*tert*-Butyl-2-methylpyridine 1-oxide (1.2 g, 6 mmol) was taken into H_2_SO_4_ (2 ml), and HNO_3_ (1.5 ml) was added at 0°C. The reaction mixture was heated at 100°C overnight and poured onto ice. The product was extracted using dichloromethane dried over MgSO_4_ and the solvent was removed under vacuum to give a colourless oil (1.1 g.73%). ^1^H NMR (400 MHz,CDCl_3_): δ 8.42 (s, 1H, H^6^), 7.33 (s,1H, H^3^), 2.50 (s, 3H, Me), 1.47 (s, 9H, ^t^Bu). ^13^C NMR (101 MHz, CDCl_3_): δ = 147.4 (C^2^), 145.7 (C^6^), 137.4 (C^5^), 131.7 (C^3^), 116.7 (C^4^), 33.6 (C(CH)_3_), 30.6 (C(CH)_3_), 16.9(CH_3_); ); ESI-LRMS (+) *m/z* 210.1 [M+H]^+^; ESI-HRMS (+) calcd for C_10_H_15_N_2_O_3_ 211.1069, found 211.1079.

**(5-*tert*-Butyl-4-nitropyridin-2-yl)methanol**

**
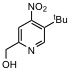
**

Trifluoroacetic anhydride (30 mL) was added to a solution of 5-*tert*-butyl-4-nitro-2-methylpyridine 1-oxide (1.6g, 7.5 mmol) in DCM (30 mL). The resulting mixture was heated at 60°C for 18 h under an inert atmosphere. After this time, the solvent was removed under reduced pressure and reaction completion to the trifluoroacetate intermediate was confirmed by ^1^H NMR analysis. The resulting bright yellow oil was stirred in a mixture of EtOH (5 mL) and H_2_O (5mL) for 1 h. The solvent was removed and the residue was taken into ethyl acetate (20ml). The organic layer was washed with dilute aqueous sodium hydroxide solution, dried over MgSO_4_ and solvent was removed under reduced pressure to yield a yellow oil (1.1g, 69 %); ^1^H NMR (400 MHz, CDCl_3_): δ 8.76 (s, 1H, H^6^), 7.27 (s, H^3^), 4.78 (s, 2H, CH_2_OH), 1.39 (s, 9H, ^t^Bu); ^13^C NMR (101 MHz, CDCl_3_): δ 161.1(C^4^), 157.5(C^2^), 150.5(C^3^), 133.5(C^5^), 114.0 (C^6^), 64.0(CH_2_OH), 34.6(C(CH)_3_), 30.5(C(CH)_3_); ESI-LRMS (+) *m/z* 211 [M+H]^+^; ESI-HRMS (+) calcd for C_10_H_15_N_2_O_3_ 211.1069, found 211.1073.

**(5-*tert*-Butyl-4 ethyl thioglycolate-pyridin-2-yl)methanol**


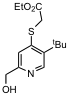


Ethyl thioglycolate (1.7 ml, 14.4 mmol) and sodium hydride ( 0.8g, 33 mmol) were taken into anhydrous dimethylformamide (3 ml), and (5-*tert*-butyl-4-nitropyridin-2-yl)methanol (1.0g, 4.8mmol) in DMF (2 ml) was added at RT and the solution was stirred for an hour. Dimethyl formamide was removed under reduced pressure and the residue was taken to dichloromethane. Inorganic salts were filtered off and solvent was removed. The crude residue was purified using silica column chromatography, eluting with a gradient starting from DCM to 2 % MeOH/DCM to yield a yellow oil (0.45 g, 30 %), *R*_f_ (5 % MeOH / DCM) = 0.4); ^1^H NMR (400 MHz, CDCl_3_): δ 8.43 (s, 1H, H^6^), 7.22 (s, H^3^), 4.68 (s, 2H, CH_2_OH),4.19 (q, *J* = 7 Hz, 2H, OCH_2_), 3.78 (s, 2H, SCH_2_), 1.49 (s, 9H, ^t^Bu), 1.24 (t, 3H, *J* = 7 Hz, CH_2_CH_3_) ; ^13^C NMR (101 MHz, CDCl_3_): δ 168.1(C=O), 157.5(C^4^), 147.9(C^2^), 145.8(C^5^), 140.0 (C^3^), 118.4(C^6^),, 64.2(CH_2_OH), 62.2 (OCH_2_) 35.4 (SCH_2_), (34.6(C(CH)_3_), 30.5(C(CH)_3_); ESI-LRMS (+) *m/z* 284 [M+H]^+^; ESI-HRMS (+) calcd for C_14_H_22_NO_3_S 284.1320, found 284.1314.

**(5-*tert*-Butyl-4 ethyl thioglycolate-pyridin-2-yl)methyl-methanesulphonate**


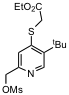


5-*tert*-Butyl-4 ethyl thioglycolate-pyridin-2-yl)methanol (450 mg, 1.60 mmol) was dissolved in THF (10 mL) and cooled to 5°C. Triethylamine (0.35 mL, 3.20 mmol) and mesyl chloride (0.20 mL, 2.40 mmol) were added dropwise to this solution. Once addition was complete, the reaction mixture was allowed to warm to RT and stirred for 2 h, before the solvent was removed under reduced pressure. The residue was treated with brine (10 mL) and extracted with DCM (2 x 10 mL). The organic layers were combined, dried over MgSO_4_, and the solvent removed under reduced pressure to yield an orange oil, which was used immediately (500 mg, 87 %). *R*_f_ (10 % MeOH/DCM) = 0.56; ^1^H NMR (400 MHz, CDCl_3_): δ 8.68 (d, *J* = 2 Hz, 1H, H^6^),7.52 (d, *J* = 8 Hz, 1H, H^3^), 5.39 (s, 2H, CH_2_OMs), 3.78 (s, 2H, SCH_2_), 3.12 (s, 3H, SO_2_CH_3_), 1.37 (s, 9H, ^t^Bu); ESI-LRMS (+) *m/z* 244.2 [M+H]^+^; ESI-HRMS (+) calcd for C_11_H_17_NO_3_S 244.1007, found 244.1020

**Di-tert-butyl 4,10-bis((5-(tert-butyl)-4-((2-ethoxy-2-oxoethyl)thio)pyridin-2-yl)methyl)-1,4,7,10-tetraazacyclododecane-1,7-dicarboxylate**


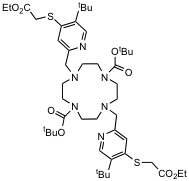


Di-tert-butyl 1,4,7,10-tetraazacyclododecane-1,7-dicarboxylate (0.3g,0.75mmol) was dissolved in acetonitrile (30ml) and (5-*tert*-butyl-4 ethyl thioglycolate-pyridin-2-yl)methyl-methanesulphonate (0.65g, . 1.87mmol) and potassium carbonate (0.22g, 1.65mmol) were added. The reaction mixture was heated at 80°C for 10h. Inorganic salts were filtered off and solvent was removed under reduced pressure to give a pale yellow oily product. The crude residue was purified using silica column chromatography, eluting with a gradient starting from DCM to 5 % MeOH/DCM to yield an oily product (330mg, 50%). *R*_f_ (5 % MeOH/DCM) = 0.2). ^1^H NMR (400 MHz, CDCl_3_): δ = 8.41 (s, 2H, H^6^), 7.26 (s, 2H, H^3^), 4.19( 2xt, J = 4 Hz, 4H, OCH_2_CH_3_), 3.96 (s,4H, SCH2), 3.83 (s, 2H, NCH_2_Py),3.74(s, 2H, NCH_2_Py) , ( 3.49-3.37 (br m, 12H, cyclen-CH_2_),2.78-2.62 (br m, 4H, cyclen-CH_2_), 1.51 (s, 18H, ^t^Bu), 1.27 (s, 18H, ^t^Bu), 1.24 (t, J= 4Hz, 6H, CH_2_CH_3_); ^13^C NMR (101 MHz, CDCl3): δ = 168.8 (CO_2_Et), 155.9 (CO_2_tBu), 146.6 (C^4^), 143.8(C^2^), 140.7 (C^5^), 120.8 (C^3^), 119.7 (C^6^), 61.9 (OCH_2_), 60.2 (NCH_2_Py), 58.2 (cyclen–CH_2_), 54.6 (cyclen-CH_2_), 52.8 (cyclen-CH_2_), 52.4 (cyclen-CH_2_), 46.3 (cyclen-CH_2_), 48.3 (cyclen-CH_2_), 35.4 (SCH_2_) 35.4 (C(CH_3_)_3_), 29.8 (C(CH)_3_), 28.5 (C(CH_3_)_3_), 14.3(CH_2_CH_3_); ESI/MS+ m/z 903.5 [M+H]+, HRMS Calcd for C_46_H_75_N_6_O_8_S_2_  903.5088. Found 903.5117

**Diethyl 2,2'-((((1,4,7,10-tetraazacyclododecane-1,7-diyl)bis(methylene))bis(5-(tert-butyl)pyridine-2,4-diyl))bis(sulfanediyl))diacetate**


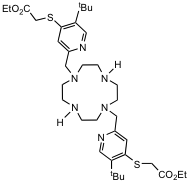


Di-tert-butyl 4,10-bis((5-(tert-butyl)-4-((2-ethoxy-2-oxoethyl)thio)pyridin-2-yl)methyl)-1,4,7,10-tetraazacyclododecane-1,7-dicarboxylate (0.33g, 0.37mmol) was taken to dichloromethane (2ml) and trifluoroacetic acid (2ml) was added and stirred at room temperature for 18h. The solvent was removed and dichloromethane (2ml) was added and removed under reduced pressure (repeated three times) to remove traces of trifluoroacetic acid. The residue was taken into water (2ml) and pH was adjusted using dilute sodium hydroxide solution to 10-11. The product was extracted using dichloromethane, dried using MgSO_4_ and solvent was removed under reduced pressure to give a clear oily product (0.25g, 96%). ^1^H NMR (400 MHz, CDCl_3_): δ = 8.44 (s, 2H, H^6^), 7.63 (s, 2H, H^3^), 4.18 (q, J = 4 Hz, 4H, OCH_2_CH_3_), 3.96 (s,4H, SCH_2_), 3.99 (s, 4H, NCH_2_Py), 3.23 (br m, 8H, cyclen-CH_2_),3.14 (br m, 8H, cyclen-CH_2_), 1.48 (s, 18H, ^t^Bu), 1.24 (t, J = 7 Hz, 6H, CH_2_CH_3_); ^13^C NMR (101 MHz, CDCl_3_): δ = 168.8 (CO_2_Et), 156.6 (C^4^), 149.8(C^2^), 143.7 (C^5^), 142.1 (C^3^), 122.53 (C^6^), 61.9 (OCH_2_), 60.2 (SCH_2_), 55.1 (NCH_2_Py), 53.5(cyclen–CH_2_), 49.1 (cyclen-CH_2_), 43.5 (cyclen-CH_2_), 35.4 (SCH_2_) 35.4 (C(CH_3_)_3_), 29.8 (C(CH)_3_), 28.9 (C(CH_3_)_3_), 14.1(CH_2_CH_3_); ESI/MS+ m/z 702.5 [M+H]+, HRMS Calcd for C_36_H_59_N_6_O_4_S_2_  703.4039. Found 703.4063

**Diethyl 2,2'-((((4,10-bis((ethoxy(methyl)phosphoryl)methyl)-1,4,7,10-tetraazacyclododecane-1,7-diyl)bis(methylene))bis(5-(tert-butyl)pyridine-2,4-diyl))bis(sulfanediyl))diacetate**


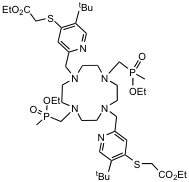


Diethyl 2,2'-((((1,4,7,10-tetraazacyclododecane-1,7-diyl)bis(methylene))bis(5-(tert-butyl)pyridine-2,4-diyl))bis(sulfanediyl))diacetate (0.3g, 0.43mmol)was dissolved anhydrous THF (25ml). Paraformaldehyde (0.21g, excess) was added and the solution was boiled under reflux over molecular sieves (4Å) using a Soxhlet condenser under argon. Methyl diethoxy phosphine (0.3g, 1.6mmol) was added and continued to heat at reflux temperature for 18hrs. Inorganic salts were filtered off, and solvent was removed under reduced pressure to give an orange oily product. The crude residue was purified using alumina column chromatography eluting with a gradient starting from DCM to 2 % MeOH/DCM to yield a yellow oil (160mg, 40%). *R*_f_ (10 % MeOH/DCM) = 0.25). ^1^H NMR (400 MHz, CDCl_3_): δ = 8.44 (s, 2H, H^6^), 7.97 (s, 1H, H^3^), 7.45 (s, 1H, H^3^), 4.17(q, J = 7 Hz, 4H, OCH_2_CH_3_), 4.07 (m, 4H, POCH_2_), 3.84 (s,4H, SCH_2_), 3.71 (s, 2H, NCH_2_ py ),3.64(s, 2H, NCH_2_py) , ( 3.07-2.94 (br m, 4H, PCH_2_), 2.78-2.58 (br m, 12H, cyclen-CH_2_), 2.49-2.38(br m, 4H,cyclen-CH_2_), 1.49 (s, 18H, tBu), 1.43 (d, J = 16 Hz, 6H, PCH_3_), 1.26 (t, J = 7 Hz, 6H, CH_2_CH_3_), 1.21(t, J= 4 Hz, 6H, CH_2_CH_3_); ^13^C NMR (101 MHz, CDCl_3_): δ = 168.8 (CO_2_Et), 156.7 (C^4^), 146.7, 146.4(C^2^), 140.6, 140.5 (C^5^), 122.8, 121.2 (C^3^), 120.7 (C^6^), 61.9 (OCH_2_),61.1(POCH2) 60.2 (NCH_2_py), 55.4 (cyclen–CH_2_), 55.0 (cyclen-CH_2_), 54.3 (cyclen-CH_2_), 53.5 (cyclen-CH_2_), 53.0 (cyclen-CH_2_), 52.2 (cyclen-CH_2_),47.3 (NCH_2_P) 35.4, 35.3 (SCH_2_) 35.6 (C(CH_3_)_3_), 29.9 (C(CH)_3_), 28.5 (C(CH_3_)_3_), 16.9 (CH_2_CH_3_), 14.3(CH_2_CH_3_); ^31^P NMR (162 MHz, CDCl_3_) δ = 53.2; ESI/MS+ m/z 942.5 [M+H]+, HRMS Calcd for C_44_H_76_N_6_O_8_P_2_S_2_  943.4720. Found 943.4741

**2,2'-((((4,10-Bis((hydroxy(methyl)phosphoryl)methyl)-1,4,7,10-tetraazacyclododecane-1,7-diyl)bis(methylene))bis(5-(tert-butyl)pyridine-2,4-diyl))bis(sulfanediyl))diacetic acid , L^1^.**


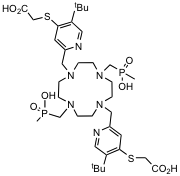


Diethyl 2,2'-((((4,10-bis((ethoxy(methyl)phosphoryl)methyl)-1,4,7,10-tetraazacyclododecane-1,7-diyl)bis(methylene))bis(5-(tert-butyl)pyridine-2,4 diyl))bis(sulfanediyl))diacetate (0.16g, 0.16mmol) was dissolved in hydrochloric acid (6M, 10ml) and heated at80°C for 18 hrs. Water was removed under reduced pressure and the residue was washed with dichloromethane to give a glassy solid (0.12g, 85%). ). ^1^H NMR (400 MHz, CD_3_OD): δ = 8.49 (s, 1H, H^6^), 8.44 (s, 1H, H^6^) 8.06 (br s , 1H, H^3^), 7.91 (s, 1H, H^3^, 4.47 (s,4H, SCH_2_), 4.37 (s, 2H, NCH_2_py),4.37(s, 2H, NCH_2_py) , ( 4.04-4.08 (br m, 4H, PCH_2_),3.94-3.42 (br m, 12H, cyclen-CH_2_3.32-2.91(br m, 4H,cyclen-CH_2_), 1.62 (s, 18H, tBu), 1.61 (d, J = 16Hz, 6H, PCH_3_); ^13^C NMR (101 MHz, CD_3_OD): δ = 174.5 (C=O), 159.7 (C^4^), 147.7, 146.4(C^2^), 142.6, 140.5 (C^5^), 125 (C^3^), 122.7 (C^6^), , 62.2 (NCH_2_Py), 55.4 (cyclen–CH_2_), 55.0 (cyclen-CH_2_), 54.3 (cyclen-CH_2_), 53.5 (cyclen-CH_2_), 53.0 (cyclen-CH_2_), 52.2 (cyclen-CH_2_), 47.3 (NCH_2_P) 35.4, 35.3 (SCH_2_), 35.6 (C(CH_3_)_3_), 28.5 (C(CH_3_)_3_; ^31^P NMR (162 MHz, CDCl_3_) δ = 30.1; ESI/MS+ m/z 830.3 [M+H]+, HRMS Calcd for C_36_H_61_N_6_O_8_P_2_S_2_  831.3468. Found 831.3465; m.p : >170°C (dec)

**[DyL^1^]**

Dy(III)Cl_3_.6H_2_O (27 mg, 0.06mmol) was added to a solution of 2,2'-((((4,10-bis((hydroxy(methyl)phosphoryl)methyl)-1,4,7,10-tetraazacyclododecane-1,7-diyl)bis(methylene))bis(5-(tert-butyl)pyridine-2,4-diyl))bis(sulfanediyl))diacetic acid (L_4_) (40mg, 0.05 mmol) dissolved in H_2_O (3 mL). The pH was adjusted to 5.5 before stirring the solution for 18 h at 80°C. After this time, the solution was allowed to cool to RT before the pH was raised to 10 using NaOH solution, causing a white solid to precipitate out. This precipitate was removed by centrifugation and the pH of the resulting solution was neutralised using HCl (1M). The solvent was removed under reduced pressure to yield a yellow solid, (30 mg, 98 %). The complex was purified using reverse phase HPLC (*t*_R_ = 7.5 min). ^1^ NMR (400 MHz, D_2_O, pD 6.9, 295K): δ = -60.15 major, -64.25 minor (^t^Bu); ESI/MS^+^ m/z 991.5 [M]^+^; HRMS Calcd for C_36_H_58_^160^DyN_6_O_8_P_2_S_2_ 988.2508. Found 988.2508

**[Gd L^1^]**

An analogous procedure to that described for **[Dy.L^1^]** was followed using Gd(III)Cl_3_.6H_2_O and solution of 2,2'-((((4,10-bis((hydroxy(methyl)phosphoryl)methyl)-1,4,7,10-tetraazacyclododecane-1,7-diyl)bis(methylene))bis(5-(tert-butyl)pyridine-2,4-diyl))bis(sulfanediyl))diacetic acid (L_4_). ESI/MS^+^ m/z 986.5 [M]^+^; HRMS Calcd for C_36_H_58_^155^GdN_6_O_8_P_2_S_2_ 983.2459. Found 983.2474. r_1p_ : 2.4 mM^-1^ **_S_^-1^** (pH 6, 1.4 T, 310 K)

HPLC : *t*_R_ = 7.5 min.
